# Deputy Surgeon-General Robert Walter Clifton

**Published:** 1911-09-16

**Authors:** 


THE death is announced of Deputy Surgeon-General Robert Walter Clifton, formerly of the Army Medical Staff. He entered the Army in 1857, immediately after qualification, and was present at the action of Sinho and the capture of the Taku Forts, receiving the medal with clasp. He attained the rank of Deputy Surgeon-General in 1887, about which time he was principal medical officer of the Curragh Brigade. He retired in 1889.

